# Biomodification Strategies for the Development of Antimicrobial Urinary Catheters: Overview and Advances

**DOI:** 10.1002/gch2.201700068

**Published:** 2017-12-27

**Authors:** Sadiya Anjum, Surabhi Singh, Lepoittevin Benedicte, Philippe Roger, Manoj Panigrahi, Bhuvanesh Gupta

**Affiliations:** ^1^ Bioengineering Laboratory Department of Textile Technology Indian Institute of Technology New Delhi 110016 India; ^2^ ICMMO ‐ LG2M ‐ Bât 420 Université Paris‐Sud XI, 15 rue Georges Clémenceau 91405 Orsay Cedex France; ^3^ Department of Urology and Pathology Sikkim Manipal Institute of Medical Sciences Gangtok Sikkim 737101 India

**Keywords:** antimicrobial, biofilms, functionalization, infection, urinary catheters

## Abstract

Microbial burden associated with medical devices poses serious health challenges and is accountable for an increased number of deaths leading to enormous medical costs. Catheter‐associated urinary tract infections are the most common hospital‐acquired infections with enhanced patient morbidity. Quite often, catheter‐associated bacteriuria produces apparent adverse outcomes such as urosepsis and even death. Taking this into account, the methods to modify urinary catheters to control microbial infections with relevance to clinical drug resistance are systematically evaluated in this review. Technologies to restrict biofilm formation at initial stages by using functional nanomaterials are elucidated. The conventional methodology of using single therapeutic intervention for developing an antimicrobial catheter lacks clinically meaningful benefit. Therefore, catheter modification using naturally derived antimicrobials such as essential oils, curcumin, enzymes, and antimicrobial peptides in combination with synthetic antibiotics/nanoantibiotics is likely to exert sufficient inhibitory effect on uropathogens and is extensively discussed. Futuristic efforts in this area are projected here that demand clinical studies to address areas of uncertainty to avoid development of bacterial resistance to the new generation therapy with minimum discomfort to the patients.

## 1 Introduction

Microbial infection from medical devices, especially from tissue contacting implants projects a severe health risk to patients who are catheterized to manage urinary incontinence and urinary retention. Nosocomial infections can reach up to any level (including kidney) and may become persistent, dangerous, and fatal. The work in this area offers a challenge to the medical fraternity to look for an appropriate alternative.^1^, ^2^, ^3^, ^4^ Urinary tract infections (UTIs) are among the most common nosocomial infections occurring in either the community or healthcare setting. 40% of all healthcare associated infections comprise of UTIs and about 80% of them are related to catheter use, resulting in significant influence on patient's morbidity and mortality.^5^, ^6^ The quantum of infections in fact depends on the duration of the catheterization. Severe infections arise from the use of indwelling urinary catheters where the bacterial colonization takes place within two weeks, which becomes more prominent with the passage of catheterization.^7^ The open catheter drainage systems may lead to almost 95% infection rate due to bacteriuria as the opening is not sealed against the entrance of outside air, whereas infection rate in a closed catheter drainage system has been found to be limited to 5%, per day of catheterization. Among the surgical site infections, catheter related urinary infection becomes more dominant in aged people requiring frequent medical care.^8^ From 5% to 10% of elderly residents on long term care facility require indwelling catheter for management of urinary voiding.^9^

The urinary catheter implantation opens up a direct route to bacterial invasion and biofilm formation by avoiding the skin barrier.^10^ The catheter contacts colonized perineum and creates friction against bladder mucosa leading to inflammatory response, thus providing a route for bacterial entry along both its internal and external surfaces.^11^, ^12^, ^13^, ^14^ The bacterial count can increase up to many folds as measured in terms of colony forming units (CFUs) within a day or so, suggesting a fast increase in their number.^15^ Moreover, the inserted catheter gets a thin protein layer as covering on its surface, providing a bioreceptive interface which attracts a large chunk of microbes to adhere and proliferate.^16^ The complications of both the short term and long term catheterization (LTC) have been reviewed here.^17^ This review aims at addressing the major issues surrounding catheter‐associated urinary tract infections (CAUTIs) among patients with LTC, rapid increase of antibiotic resistance, as well as the current and future approaches to design antimicrobial using synthetic and natural biomaterials to combat CAUTIs.

## 2 Biofilm Formation and Microbial Communication

**Figure 1:**
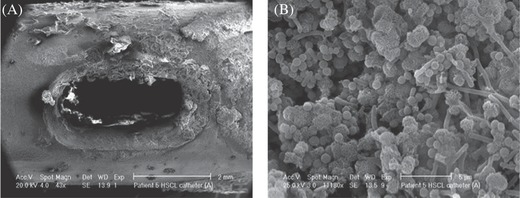
A) Encrustation around the eyelet of a catheter removed from a patient after just 5 d, and B) bacilli, cocci, and microcrystalline aggregates of calcium phosphate in the crystalline biofilm around the eyelet of the catheter. Reproduced with permission.^19^ Copyright 2009, Springer.

Long term indwelling bladder catheterization in patients causes major complication due to the encrustations (usually composed of calcium and magnesium phosphates) of uropathogens on the catheter surfaces. Colonization of these crystalline deposits by bacteria and entrapment of the crystals within the bacterial polysaccharide matrix result in the formation of a biofilm (**Figure** **1**). Crystalline deposit on catheter lumen can block the functions of catheter in terms of bladder distension, reflux of urine to the kidneys, or leakage around the catheter. If the blocked catheter is not changed, it causes serious complications in patient such as pyelonephritis, septicemia, and trauma to the bladder mucosa.^18^, ^19^, ^20^

There are multiple strains of organisms associated with CAUTIs, both gram‐positive and gram‐negative bacteria and occasionally fungi like *Candida* species. Of the bacterial species, the major ones involved in biofilm formation are *Escherichia coli* (*E. coli*), *Proteus mirabilis* (*P. mirabilis*), *Klebsiella pneumoniae* (*K. pneumoniae*), *Pseudomonas aeruginosa* (*P. aeruginosa*), *Enterococcus faecium*, *Staphylococcus aureus* (*S. aureus*), and coagulase negative *Staphylococci*.^21^, ^22^ These biofilm forming agents can enter the urinary tract in catheterized patients by three major routes comprising of intraluminal, extraluminal, and periurethral routes. Intraluminal route is a predominant route in males, suggesting an exogenous source. Microorganisms enter through the lumen of the urinary catheter and move into the bladder.^23^, ^24^ The organisms that access via the intraluminal route may originate from the skin of the patient as its source or may be iatrogenic, that is, arising from a healthcare worker.^25^ The extraluminal route follows where the organisms are primarily endogenous in nature and emerge from the patient's own gastrointestinal tract gain entry to the urinary tract. This is especially recurrent in patients where cleaning of the perineum, distal urethra is inadequate and lack of adequate asepsis has been used.^26^, ^27^, ^28^, ^29^ However, periurethral route of entry is especially prime in catheterized women. Subsequently, organisms enter the urinary tract from the external surface of the catheter in the mucous sheath present between the catheter and urethral mucosa.^23^ Contaminating organisms from the periuretheral area may also ascend along the external surface of the catheter and establish infection.^25^, ^30^

Once inside the catheter, microbial biofilm is a major impediment to the use of indwelling medical devices.^31^ The formation of biofilm generally consists of following main steps: The first step in biofilm formation is the attachment of the bacteria to the material surface. The microbial adhesion is governed by the physicochemical nature of the catheter, such as surface polarity and charge density, reversibly by weak van der Walls forces and hydrogen bonding. Biofilm, therefore, leads to the chronic and persistent infections which would not be cured by normal antimicrobial therapy due to protective layer surrounding them. If we look at the mechanism of the biofilm formation, the very first step of the insertion of the catheter into the urinary system plays a very crucial role. The catheter undergoes conditioning within the urinary surroundings so that the deposition of urinary components, such as electrolytes, organic components, and proteins on the catheter surface takes place.^32^ This makes the catheter surface bioreceptive in nature where microbes can find a very favorable environment to attach on the catheter surface. Infections are caused primarily due to the microbial invasion at the catheter insertion and tissue at the catheter interface. Bacteria subsequently multiplies and excrete extracellular matrix so that a loosely crosslinked 3D structure is formed with fluid channels to allow exchange of nutrients.^33^ Once attached, bacteria produces the hydrated polysaccharides and protein matrix termed as exopolysaccharides (EPS) which act as the protective layer for the bacterial existence.^31^, ^34^ Bacteria such as *P. aeruginosa* and *K. pneumoniae* can produce copious amounts of EPS that result in the formation of mucoid biofilms that can occlude catheter lumens.^35^

One of the major problems with biofilm formation is the microbial resistance against conventional antibiotics, leading to the failure of the protective approach in patients.^36^, ^37^ Microorganisms embedded in biofilms under encasing slimy EPS matrix display increased antibiotic resistance compared to their planktonic state. Some strains of pathogens become resistant to many antibiotics and other therapeutic drugs, which is referred to as the multidrug resistance.^38^ In the race of fast pace development of antimicrobial agents, superbugs (microbes) are becoming winners and the new drugs are rendered ineffective.^39^ This is due to slow or very little diffusion of the drug within the biofilm domain. Thus, looking at the complexity of the bacterial infection and biofilm formation in CAUTIs, it becomes extremely important to develop and design the biomaterial which is efficient to control microbial infection on medical devices.^40^, ^41^, ^42^, ^43^

## 3 Strategies for Developing Antimicrobial Catheter Surface

**Figure 2:**
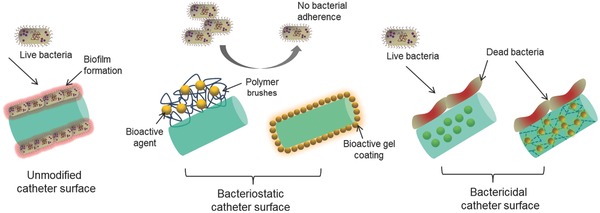
Schematic representation depicting the bacteriostatic and bactericidal activity of antimicrobial catheter surfaces.

Microbes influencing the infections and biofilm formation have led to enormous research dealing with the development of the material which is inherently antimicrobial in nature. The approach involves the material designing so that it behaves as antiseptic, bacteriostatic, and bactericidal in nature, i.e., the material resists the adherence and propagation of microbes due to the linking of the catheter surface with bioactive agent. It is desirable to have a proactive approach by preventing biofilm formation rather than attempting to eliminate them by creating unfavorable conditions for bacteria. Efforts are being made in both the directions where the surface is made infection resistant by coating or immobilizing the bioactive components on it. The surface functionality with antimicrobial agent may be the direct result of interaction of bioactive component with the surface by ionic bond or covalent bond or hydrogen bonding. Although, there are different catheters for urinary systems, silicon catheter is the most widely used one due to the fact that they resist encrustation much better than latex ones.^5^ At the same time, polyurethane catheters are considered to be one of the most biocompatible materials because of their excellent properties such as toughness, fatigue resistance, and durability. Alternatively, the bulk modification of the materials may also be carried out so that whole matrix becomes antimicrobial in nature and controls bacterial infection. However, there has not been any such material which would offer complete inhibition of bacterial adhesion (zero tolerance) where bacteria are killed once it comes in contact to material surface. Mainly, surface properties of materials including surface morphology, surface functionality, polar interactions, surface charge density, and hydrophobicity/hydrophilicity nature play a very important role in bacterial adhesion.^44^, ^45^, ^46^, ^47^, ^48^ Taking this into account, modification of the catheter surface is primarily accomplished by employing four major strategies; functionalization, coating, drug impregnation, and blending. Functionalization and coating are effective in the development of bacteriostatic surfaces, whereas drug impregnation and blending give rise to bactericidal nature in the material as depicted in **Figure** **2**.

The above strategies employed for catheter surface modification make use of antibiotics, but looking at the complex scenario of biofilm formation, it is very difficult to treat the infection once biofilm develops, due to the negligible permeability of traditional antimicrobial agents and their inability to act against microbes.^49^, ^50^ In this scenario, nanoantibiotics, which are composed of nanomaterials, have gained increasing attention. In comparison to conventional antibiotics, nanoantibiotics are retained in the body for a much longer duration than small molecule antibiotics, are cost‐effective, stable during prolonged storage time, can withstand harsh conditions such as sterilization at high temperatures, where conventional antibiotics fail to exhibit their effect.^51^ Particularly, nanosized Ag, zinc oxide, aluminum oxide, titanium dioxide, gold, and copper as well as carbon nanotubes have been reported to be efficacious in deactivating various microorganisms and find applications in catheter coating in hospitals.^52^, ^53^, ^54^, ^55^, ^56^ Use of traditional antibiotics and nanoantibiotics during fabrication of antimicrobial catheters using different strategies has been described in detail in the following sections.

### 3.1 Catheter Surface Functionalization

**Figure 3:**
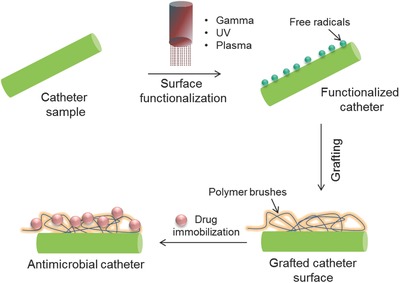
Schematic representing the strategies for catheter functionalization by grafting using different sources (gamma, UV, and plasma).

Functionalization of the catheter surface by antimicrobial agents is a promising strategy against pathogen colonization. Bioactive molecules may be immobilized either by covalent attachment to activated surface groups or through hydrophobic/ionic interactions. Covalent immobilization becomes salient for binding of molecules that do not adsorb at all, adsorb very weakly, or adsorb with inappropriate orientation and may result in enhanced biomolecule activity, reduction in nonspecific adsorption, and greater stability. But, one of the classical problem that arises is the effective binding of these bioactive agent to the surface, as common catheters such as silicone, polyethylene, polyurethane, and latex are hydrophobic in nature and do not have ability to bind in a stable manner. Therefore, catheter surface needs functionalization in such a manner that polar or ionic groups are introduced on its surface for interaction with the bioactive agents. Functionalization involves the use of plasma, gamma, and ultraviolet radiations to carry out the modification process as shown in **Figure** **3**.

The process is based on generating polymer brushes via functionalization. The innovation in this approach is that the material may be designed in such a way that it is the best suited for inhibiting bacterial adhesion or killing them once they are in contact with these brushes or both. Plasma functionalization is a very effective method to functionalize material surfaces where desired functional groups may be incorporated on the surface so that subsequent immobilization of a bioactive component may be accomplished.^57^ Low temperature radio frequency plasma is one of the techniques that has been largely employed to generate different functional groups on the material surface, depending on the nature of the gaseous medium. While ammonia plasma introduces amino functionality, carbon dioxide leads to the generation of carboxyl groups, depending on the plasma treatment conditions. The advantage of the plasma treatment is that the functionalization is limited to a few nanometers on the catheter surface due to low energy levels. As a result, the bulk properties of the material remain unaffected. Ethylene diamine (EDA) plasma has been used to develop a layer with amino groups followed by the deposition of alginate layer.^58^ Although, the plasma processing conditions such as plasma power, plasma exposure time, contact angle, and bacterial adhesion were investigated, alginate coating reduced the *E. coli* adhesion more than the EDA containing surfaces. However, a complete inhibition of bacterial growth on catheter surfaces could not be achieved. As, one can see that the functionality created by plasma treatment remains low, which needs to be enhanced for better bonding and efficiency of the biomaterial. Lim et al. tethered CWR11 antimicrobial peptides (AMPs) on a silicon surface by plasma activation which holds potential for the development of peptide based antimicrobial catheters.^59^

Apart from AMPs and antimicrobial polymers, antimicrobial hydrogels have also emerged as an essential platform to combat infections associated with medical devices and primarily conjugate to the surface by covalent linkages. Liu et al. aimed at creating antifouling antimicrobial hydrogels, which can be formed in situ and may be easily applied onto implants.^60^ Here, polycarbonate containing quaternary ammonium groups were chemically incorporated into polyethylene glycol (PEG) hydrogel networks via Michael addition chemistry and these hydrogels possessed strong antimicrobial activity against multidrug‐resistant gram‐positive and gram‐negative bacteria as well as negligible toxicity toward mammalian cells. These hydrogels were grafted onto silicone rubber, a material used for catheters and effective antifouling (100% reduction in *Candida albicans* (*C. albicans*) colonies) and antimicrobial activity (98–100% reduction in *E. coli* and *S. aureus* colonies, respectively) on the material were observed that have also been supported by bacterial and fungal adherence studies. Similarly, Kara et al. covalently immobilized chitosan, a well‐known antimicrobial hydrogel onto polyurethane (PU) films and examined their antimicrobiality against *S. aureus* and *P. aeruginosa* 61 in terms of reduction in bacterial concentration with respect to control, which was found to be 2.5 and 4.2 log CFU mL^−1^ for *P. aeruginosa* and *S. aureus*, respectively. The results reveal higher activity against *P. aeruginosa* as compared to *S. aureus*. The reason behind this could be bacteria surface polarity as it is known that the outer membrane of *P. aeruginosa* consists of mainly lipopolysaccharides containing phosphate and pyrophosphate groups which enhance negative charge density on the surface, hence leading to greater attraction to the positive chitosan surface compared to *S. aureus* which possesses peptidoglycan membrane.

**Figure 4:**
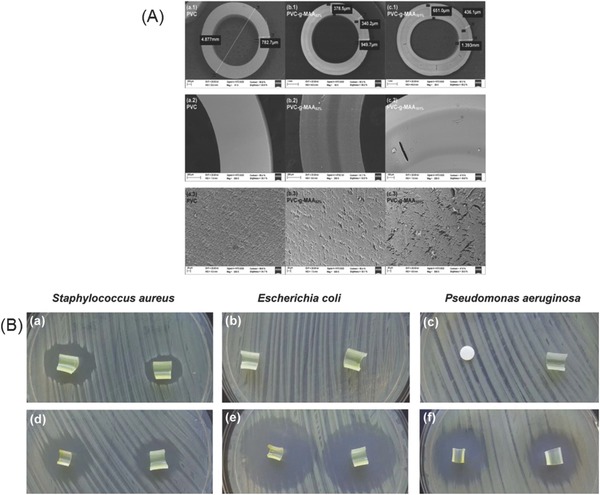
A) Scanning electron microscopy (SEM) images of cross‐sectional area of pristine, grafted, and after swollen in phosphate buffered solution (PBS). B) Inhibition zones in *S. aureus*, *E. coli*, and *P. aeruginosa* cultures of PVC‐*g*‐MAA catheters (a–c) benzalkonium chloride‐loaded PVC‐*g*‐MAA graft catheters. (d–f) Ciprofloxacin‐loaded PVC‐*g*‐MAA graft catheter (piece on the left on the agar plate) and 85% graft catheter (piece on the right). Reproduced with permission.^69^ Copyright 2017, Elsevier.

Both the high energy radiation and plasma have been used to graft different monomers on polymer surfaces.^62^, ^63^ Compared to the chemical methods of immobilization, gamma radiation induced grafting is advantageous since it is applicable to a wide variety of polymer–monomer combinations and does not require chemical initiators which leave residues behind as the impurity. The creation of polymer brushes on a polymer surface provides a unique opportunity to develop antifouling surfaces via grafting process.^64^ For instance, PEG or polyethylene oxide (PEO) brushes have been designed as antiadhesion layers that can be created on all the hydrophilic and hydrophobic surfaces.^65^ Almost complete inhibition (94% reduction in bacterial attachment) against *P. aeruginosa* has been reported by PEO brushes on silicon surfaces.^66^ Recent reports demonstrate that the functionalization of biomedical devices with smart polymers applying gamma radiation is an efficient tool for preventing biofilm formation.^62^, ^67^, ^68^ Poly(vinyl chloride) (PVC) catheter was functionalized by the radiation‐grafting of pH‐responsive methacrylic acid (MAA) to create poly(methacrylic acid) brushes. Functionalized surface showed enhanced capability to immobilize ciprofloxacin in presence of benzalkonium chloride (as surfactant) to improve the sustained release of the drug in both acid and alkaline pH for one week at very low concentration of 0.015 mg mL^−1^. Remarkable zone of inhibition was observed in functionalized catheter against *E. coli*, *P. aeruginosa*, and *S. aureus* and results are shown in **Figure** **4**,^69^ where the synergistic effect of benzalkonium chloride and ciprofloxacin is prominent.

The graft polymerization of an appropriate monomer onto plasma treated surface offers an attractive route to the high density functionalization on the surfaces.^70^ The carboxyl content after acrylic acid graft functionalization may reach up to 600 mm cm^−2^ as observed in the case of polypropylene.^71^ A study attempted to covalently link PEG brushes to silicone surface with subsequent combination of PEGylated silicone with triclosan so that antimicrobial nature can be introduced into catheter. Triclosan is known to be very effective antimicrobial agent and helps in preventing the crystalline biofilm induced blockage of the urinary bladder.^72^ Even 1% triclosan is effective in controlling the formation of biofilm that can help in the reduction of catheter‐associated urinary tract infection. However, it was observed that the PEG helps in enhancing the antimicrobial activity to 70 d as compared to 49 d without PEG.^73^ The biofilm formation has been resisted by these surfaces against *S. aureus*. Using similar approach, antibiotic eluting materials have also been developed by conjugating peptides with hydrophilic polymer brushes which exhibit biofilm resistance as well, depending on the nature of the peptide tethered on the surface by slow and prolonged drug release.^41^ Subsequently, Farrag et al. carried out the grafting of glycidyl methacrylate on PU catheter surface. The grafted surface carries epoxy groups which were reacted with amino group of gentamicin so that a covalently linked antibiotic may be developed.^74^ The modified material acquired antimicrobial and antiadherent properties against *P. aeruginosa*, *Acinetobacter baumannii*, *K. pneumonia*, and *Candida tropicalis* (*C. tropicalis*) and showed high as 23‐fold–512‐fold of the minimum inhibitory concentration (MIC) of the gentamicin. AMPs have been immobilized on polydimethyl siloxane and urinary catheter surfaces by prior functionalization using allyl glycidyl ether polymer brushes.^49^, ^75^

### 3.2 Coating on Catheter Surface

**Figure 5:**
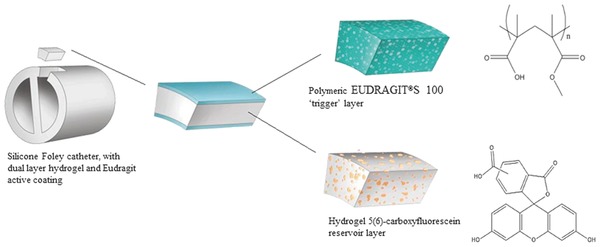
Schematic illustration of dual‐layered polymeric architecture for pH‐triggered release of 5(6)‐carboxyfluorescein. Reproduced with permission.^82^ Copyright 2016, Elsevier.

Coating of a polymer on biomaterial surface has been used to develop antimicrobial surfaces. Both the natural as well as synthetic polymers may be used for coating.^76^ Antimicrobial agents such as furanones, antibiotics (ciprofloxacin, ofloxacin, norfloxacin), a combination of antibiotics (minocycline with rifampin), have shown encouraging results for the eradication of bacterial colonization of urinary catheters.^77^, ^78^, ^79^, ^80^ Kowalczuk et al. coated small specimens of latex siliconized catheter pieces with an antibiotic sparfloxacin conjugated with heparin for the antimicrobial prevention.^81^ Assessment of antimicrobial activity of antibiotic modified catheter against *S. aureus*, *Staphylococcus epidermidis* (*S. epidermidis*), and *E. coli* strains was done using inhibition zone and diffusion assays as well as biofilm test which presented an antibacterial activity against all tested bacterial strains for at least a period of one month. Hydrogels have recently gained enormous attention as an interesting material for antimicrobial coatings. Milo et al. reported a dual‐layered pH‐responsive hydrogel surface coating for urinary catheters. The dual layer is a sandwich structure of poly(vinyl alcohol) (PVA) layer, contains the self‐quenching dye carboxyfluorescein as lower layer and this is capped by an upper layer of the pH responsive polymer poly(methylmethacrylate‐*co*‐methacrylic acid) (Eudragit S100s). The coating was engineered to provide a visual response following a pH trigger, which correlates with an early warning of urinary catheter blockage as a result of *P. mirabilis* infection (**Figure** **5**).^82^

Ahearn et al. reported that adhesion of bacteria to the catheter surface was reduced when silver (Ag) based hydrogel catheters were used, in both the cases of gram‐positive and gram‐negative bacteria.^83^ Hydrogel catheters are available in the market, for example, SYMPACATH as well as BRILLANT (Aquaflates) are being sold by Teleflex Medical. Effectiveness of the antimicrobial activity of a Bardex I.C. catheter, a hydrogel latex foley catheter with inner and outer surfaces coated with a monolayer of Ag metal has been evaluated, resulting in the reduction of the level of infection associated with UTI.^84^ Ag is the most efficient broad spectrum antimicrobial agent investigated so far and has been found highly effective against various microbes in dressings, sutures, and catheters.^85^, ^86^, ^87^, ^88^ Ag alloy/hydrogel‐coated catheters were introduced in 2000, and since then it has been investigated on different catheters.^89^ The reduction in the rate of CAUTIs ranged from 27% to 73% in such catheter.^89^, ^90^, ^91^ Hydrogels have unique property of high degree of hydration in water due to polar and hydrophilic functionality along the structure. Ionic hydrogels show much higher swelling as compared to the nonionic ones and may also offer antimicrobial nature. The hydrogel‐coated surfaces provide soft and slippery surfaces while wet which helps in minimizing the damage to the urethral mucosa.^84^

**Figure 6:**
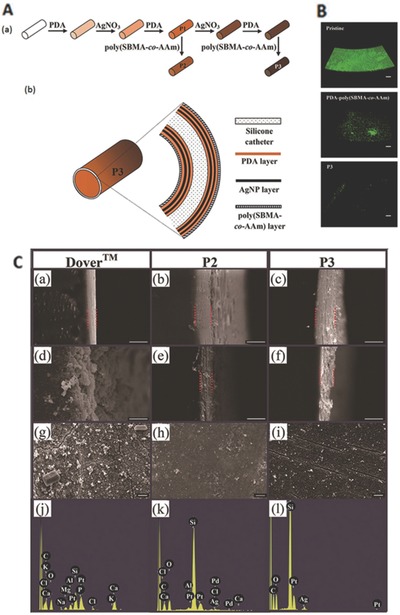
A) Schematic diagram illustrating the (a) steps for modifying a silicone catheter surface and (b) structural layers of a P3‐coated catheter. B) Confocal laser scanning microscopy images (volume view) of a *P. mirabilis* biofilm on the intraluminal surface of pristine, PDA–poly(SBMA‐*co*‐AAm)‐,and P3‐coated catheter segments after incubation in culture medium containing 105 cells mL^−1^ for 24 h. Scale bars represent 100 mm. C) Scanning electron microscopy images of the (a–f) cross‐section of the intraluminal coating and (g–i) intraluminal surface; (a–c) before the encrustation test, (d, e, g, h) after 7 d of encrustation test, and (f and i) after 40 d of encrustation test; (j–l) energy dispersive X‐ray spectra of the surfaces shown in (g–i), respectively. (a, d, g, and j): Dover Ag‐coated catheter; (b, e, h, and k): P2‐coated catheter; and (c, f, i, and l): P3‐coated catheter. The Si signal from the silicone surface in the P2‐coated and P3‐coated catheters remained prominent unlike the Dover Ag‐coated catheter which showed strong Ca and P signals. Scale bars represent 10 mm. Reproduced with permission.^92^

In a study, multilayer comprising of silver nanoparticles (AgNPs) with polydopamine (PDA) and antiadhesive poly(sulfobetaine methacrylate‐*co*‐acrylamide) poly(SBMA‐*co*‐AAm) was coated on catheter that exhibited reduced biofilm formation and resisted encrustation in artificial urine (**Figure** **6**).^92^

The antiadhesive layer prevents the deposition of a conditioning film and ensures free diffusion of the Ag from the coating. While, the other layer can reduce bacterial adhesion and biofilm formation of *P. mirabilis*, *P. aeruginosa*, and *E. coli* compared with that on the pristine silicone catheter. The rate of Ag release can be sustained at a high enough level to kill *P. mirabilis* in urine, the presence of Ag on a urinary catheter is insufficient to ensure that encrustation can be inhibited since the surface can be rapidly covered by crystalline deposits.^93^ The effectiveness of Ag‐coated silicone urinary catheter in preventing infections may be different for different systems.^86^, ^88^ This may be due to the difference in chemical nature of that catheter, which may influence interaction with Ag compound. Silvercoat is one such example of commercial catheter manufactured by Covalon and is based on silicone for developing urinary catheter. Ag alloy and Ag oxide have been used to create a layer in latex catheter which would lead to infection control during catherization.^94^ However, in a broader scene, Ag has been observed to prevent adhesion and growth of bacteria, *E. coli* and *P. aeruginosa*.^95^, ^96^ Although, Ag salts may be directly used to coat the surface, nanosilver has recently gained enormous momentum toward the generation of an effective surface against microbes due to serious problem associated with metallic salts in terms of the leaching of the agent during use and hence leading to the toxicity of the tissue.^97^, ^98^, ^99^, ^100^, ^101^ The binding of nanosilver by one way or the other becomes very important so that the leaching of the Ag may be prevented. One way is to develop a functional nanoparticle which would be able to bind the catheter surface. This may be accomplished by synthesizing nanoparticle in the vicinity of a functional organic molecule or by in situ nanoparticle formation within a polymeric nanogel.

Development of nanosilver nanohydrogels have resulted in interesting nanoparticles where the nanosilver remains entrapped within the nanogels and hence cannot escape the gel matrix.^102^ Ag release is therefore confined to the Ag ion diffusion across the hydrogel layer. This is where the use of hydrogel–Ag combination has been projected for the coating of catheter. The electrostatic interactions of the polymer with nanosilver helps in keeping the nanoparticle confined to the surface.^67^, ^102^, ^103^ The hydrogel matrix would bind the Ag but would exhibit slow release of Ag ions that proceeds across the hydrogel layer. A coating of gentamicin and poly(ethylene‐*co*‐vinyl acetate)/PEO has been observed to offer sustained release for a week (at concentration of 250 mg in 200 µL), suggesting the importance of hydrogel in coating technology.^104^ Coating of the functional nanogels has been carried out on silicone catheter surface by contacting the catheter with nanogel solution in acetone so that the catheter surface swells a bit and allows the diffusion of the nanoparticle within the surface layers. Composites with Ag have also gained increasing interest in recent years for their use as antimicrobial coatings. Sadeghi fabricated zinc oxide (ZnO) and Ag nanocomposites, which when coated on PVC significantly inhibited the growth of *S. aureus*. In addition to this, ZnO/Ag nanocomposites caused death of *S. aureus* by inducing thiol depletion.^105^ Zhou et al. designed Ag–chitosan complex and reacted it with clay to form a clay–chitosan–Ag composite which possessed excellent antibacterial properties and also has the potential to be used as a material for a sustained drug release system in indwelling urinary catheters.^106^ Further, polydimethyl siloxane/clay–chitosan–Ag nanocomposite was synthesized using an intercalation reaction and its antibacterial action was tested against *E. coli*, *P. aeruginosa*, *S. aureus*, and *C. albicans*. A study reports that thin films of Ag, plasma polymerized aniline (PPAni), and Ag–PPAni nanocomposite deposited over the surface of the latex Foley's catheters with the help of pulsed DC magnetron sputtering process acted synergistically against methicillin‐resistant *staphylococcus aureus* (MRSA) and *E. coli*.^107^, ^108^, ^109^, ^110^ In addition to nanoAg and its composites, ability of other nanoparticles to prevent catheter colonization has also been researched upon. Lellouche et al. investigated the catheter modification with the anti‐biofilm coating of magnesium fluoride (MgF_2_) nanoparticles on both inner as well as outer layer using sonochemical process, where both reduction and coating process proceeded simultaneously. The coating consisted of spherical nanoparticles of 25 nm with a thickness varying from ≈750 to 1000 nm on the inner walls and from ≈450 to ≈580 nm for the outer wall. The amount of MgF_2_ nanoparticles deposited varies from 0.021 (±0.003) to 0.010 (±0.005) mg cm^−2^ for the inside and outside walls, respectively. The coated catheter prevented biofilm formation of *E. coli* and *S. aureus* and shows remarkable antimicrobial activity. After 24 h, reduction in the biofilm viability on the outside wall was observed to be 84% and 85% on the inside wall, compared to the uncoated samples. Whereas, *S. aureus* biofilm viability exhibited reduction of 76% on both the inside and outside walls after 24 h. The effect was prominent up to 7 d where the reduction on the outside wall of both *E. coli* and *S. aureus* biofilm viability was ≈16%, and the inside wall showed a reduction of ≈20% in both bacteria.

The potential cytotoxicity of MgF_2_ nanoparticles was evaluated using human and mammalian cell lines and no significant reduction in the mitochondrial metabolism was observed, whereas, no sign of apoptosis such as the appearance of apoptotic bodies was observed.^111^

Photocatalytic disinfection is one of the most investigated approaches toward the development of infection free environment for which application of photodynamic therapy is an effective technique. Light‐activated antimicrobial agents also termed as photosensitizes show antimicrobial properties after getting activated with light of specific wavelength when coated on catheter surfaces. Sekiguchi et al. investigated the efficacy of the antimicrobial activity of titanium dioxide (TiO_2_)‐coated catheters for clean intermittent catheterization for which the catheters filled with bacterial suspensions of *E. coli*, *S. aureus*, *P. aeruginosa*, and *Serratia marcescens* (*S. marcescens*) were illuminated with UV‐A light. The survival rate of all bacterial suspensions decreased to a negligible level within 60 min of illumination. Further, the clinical trials with 18 patients who used this catheter for four weeks showed the rate of positive bacterial culture of the tips of TiO_2_‐coated catheter to be 20% versus 60% for conventional catheters.^112^ Also, the combination of antimicrobial Ag nanoparticles and photocatalyzed biocidal activity of TiO_2_ on catheter surfaces has been studied by Yao et al., where reduction in the viable count of *E. coli* to negligible levels within 3–5 min irradiation was observed in comparison to nonirradiated surface.^113^ The deposition of both TiO_2_ and Ag ions was carried out by dip coating method, which is induced by photocatalysis. He further examined the application of self‐sterilizing, self‐cleaning, and long‐lasting bactericidal property of Ag/TiO_2_ nanocomposite thin film‐coated silicon catheters under UV light illumination. The film was deposited on both the inside and outside walls of catheters and self‐cleaning property of Ag/TiO_2_ catheters was demonstrated by measuring photocatalytic degradation of methylene blue (MB) dye under UV light irradiation.

**Figure 7:**
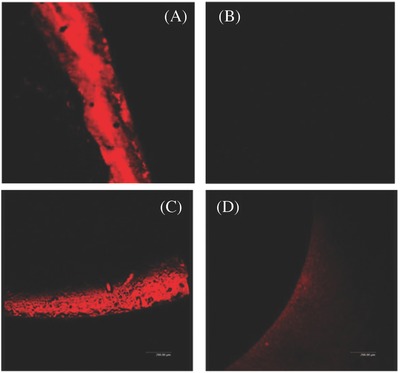
Confocal laser scanning microscopy image of A,C) control after 16 h and 7 d, B,D) JUC spray catheter after 16 h and 7 d, respectively. Reproduced with permission.^116^ Copyright 2012, BioMed Central.

Coating of natural polymers such as chitosan or combination of chitosan and heparin has also been carried out.^114^ Salicylic acid has been a bioactive component in several coating compositions and offers antimicrobial nature in urinary coatings as well.^115^ A photocurable composition has been prepared by reacting salicylic acid with acrylate monomer for coating on urinary catheter followed by UV exposure to polymerize and cure the coating. Salicylic acid is released due to ester bond cleavage when the coating comes in contact with the urine and helps in preventing the infection. He et al. investigated the modification of siliconized latex urinary catheters with JUC, commercially available antimicrobial nanospray. After 16 h of culture in *E. coli*, bacterial biofilm formation was observed on the surface of samples from control group, while the one treated with spray were free from biofilms (no growth was observed). Also, after the 7 d of catheterization, urine samples were collected for bacterial culture and notable difference was observed in the bacteriuria incidence between the treated and control group that was found to be 4.52% versus 13.04%, *p* < 0.001, respectively, as shown in **Figure** **7**.^116^

Ghanwate et al. carried out the coating of various bioactive compounds, such as DNAse enzyme, ceftazidine, ceftriaxone, cisplatin, and heparin by using 1% solution of these bioactive components by keeping it in the catheter lumen for 24 h.^117^ Biofilm formation of bacterial isolates from urinary catheters has been determined by tissue culture plate method. It was observed that biofilm was produced by *P. aeruginosa* in just 2 d in the control catheter (uncoated). But interestingly, the biofilm formation was prolonged for 14 d in ceftazidime, 8 d in ceftriaxone and cisplatin, 6 d in heparin, and 5 d in DNAse treated catheters. Koseoglu et al. carried out a systematic study on latex–silicone balloon catheters by infusing them with four antibiotics (ciprofloxacin, cefuroxime, gentamicin, and trimethoprim) to evaluate formation of biofilm of *E. coli* on the catheter surface.^118^ After initial attachment on catheter in 4–12 h, biofilm layers were observed at 12–24 h in infected groups, while the process was delayed up to 4–7 d with antibiotic treated groups. Along with single coat systems, multicoat systems comprising of layer‐by‐layer coating has emerged as an important surface coating technique to form antimicrobial films on biomedical devices which can sustain against infection for long duration.^119^, ^120^

### 3.3 Direct Drug Impregnation Process

**Figure 8:**
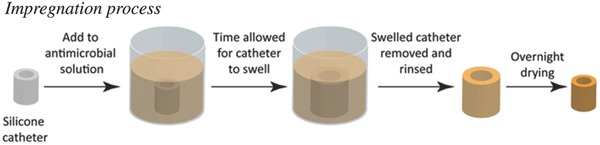
Impregnation and elution of antimicrobials from catheter. Schematic depiction of the method used to produce antimicrobial catheters. Catheters were added to a solution of antimicrobials and given time to allow the solvent to swell the catheter (1 h). Catheters were then removed from solution and allowed to dry overnight, whereupon they returned to their original size. Reproduced with permission.^121^ Copyright 2015, Elsevier.

Modification of catheters can be carried out by direct impregnation of antibiotics and other antimicrobial agents into it, where the extent of modification is dependent on the catheter material, the type, and quantity of the impregnating agents. It is a type of adhesion based upon filler matrix interface where a single homogeneous material is designed holding antimicrobial properties. The process is based on swelling of the catheter in drug solution to encapsulate the drug, subsequently followed by evaporation of the solvent, leaving behind drug impregnated catheter. Fisher et al. explained the impregnation of antimicrobial agents into Foley catheters by swelling process.^121^ Foley catheter segments were immersed in the drug solution (rifampicin, triclosan, and sparfloxacin) for certain interval of time, during which the silicone swelled to approximately twice its volume. Residual drug and solvent were removed and overnight drying was carried out. With the evaporation of the solvent, the catheters returned to their previous dimensions leaving the antimicrobials distributed evenly throughout the catheter as represented in **Figure** **8**.

Noimark et al. used PVC catheter to incorporate MB and gold (Au) nanoparticles into it, via a swell–encapsulation–evaporation method. MB and gold nanoparticle embedded PVC catheter was then irradiated with a laser light source of wavelength 660 nm, exhibiting antimicrobial activity against MRSA and *E. coli*.^122^ Results demonstrated that MRSA was highly sensitive toward the photosensitizer and nanoparticle integrated polymer, which showed 43.5 log10 reduction in the viable count after 5 min of irradiation process, in comparison with *E. coli* where a reduction of 2 log10 in the viable count was observed after irradiation for similar duration.

Silicone catheter has been impregnated with a combination of chlorhexidine and triclosan and its efficacy in providing infection resistance was evaluated by use of an in vitro model of the urinary tract.^123^ The catheter's efficacy was compared with that of the catheter impregnated with chlorhexidine, silver sulfadiazine, and triclosan and the nitrofurazone‐coated catheter and the results showed that catheter impregnated with chlorhexidine and triclosan prevented colonization with *S. aureus*, *E. coli*, *Enterobacter aerogenes*, *K. pneumoniae*, *P. mirabilis*, *Enterococcus faecalis* (*E. faecalis*), and *C. albicans* for 20–30 d or longer, compared with 4–10 d for the catheters impregnated with chlorhexidine, Ag sulfadiazine, and triclosan and for the nitrofurazone‐coated catheters. Similarly, Darouiche et al. examined the antimicrobial activity of silicone Foley catheters with minocycline and rifampin impregnated into it.^124^ The steady leaching of the antibiotics from functionalized catheter was observed for 21 d and the release of minocycline and rifampin were undetectable in serum and urine samples. The total amounts of the antimicrobial agents that could be extracted from an antimicrobial impregnated bladder catheter constitute very small fractions of the daily systemic doses of minocycline and rifampin in adults (22.3/200 mg = 11.1% and 16.4/600 mg = 2.7%, respectively). The antibiotics impregnated catheter reduced local bacterial colonization and provided broad‐spectrum antimicrobial activity against all tested urinary pathogens, including gram‐negative bacilli, gram‐positive cocci and *C. albicans*.

**Figure 9:**
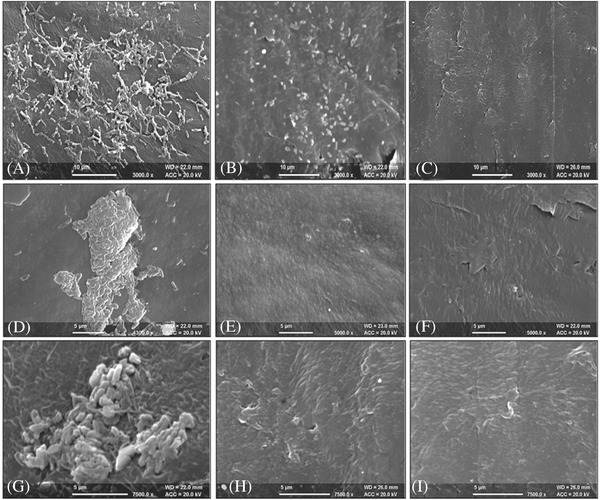
SEM images of biofilm colonization on the control, group A [2% (w/v) CIP and 5% (w/v) AZM] and group B [3% (w/v) CIP and 6% (w/v) AZM] impregnated catheters. Control catheters showed a dense biofilm of *P. aeruginosa* at A) 24 h, D) 48 h, and G) 72 h. Group A catheters revealed disrupted biofilm cells at B) 24 h; no bacterial cells at E) 48 h and H) 72 h. Biofilm colonization was completely prevented on the surfaces of group B catheters at C) 24 h, F) 48 h, and I) 72 h. Reproduced with permission.^127^ Copyright 2016, Taylor and Francis.

Ping et al. compared the antimicrobial action of levofloxacin impregnated catheters and PVC catheters against *P. aeruginosa*. Both were singly implanted subcutaneously in mice.^125^ After 1 d of in vivo implantation, three of eight catheters of levofloxacin catheter group were culture positive for *P. aeruginosa*, whereas for the PVC, eight catheters were all culture positive. No inflammation or abscess formation was found in surrounding tissues, whereas purulent secretion was found in PVC catheters and abscess formation in surrounding tissues. Fong et al. investigated the advantages of polyurethane nanocomposites (PUNCs) incorporated with an antimicrobial agent chlorhexidine diacetate for sustained release in biomedical devices like catheters.^126^ The fabricated PUNCs were incorporated with organically modified silicate (OMS) nanoparticles along with chlorhexidine diacetate using a solution‐cast method and its antimicrobial activity against *S. epidermidis* was assessed in urinary tract infection model in the urinary tract model. Drug‐release profiles demonstrated prolonged drug release instead of burst release achieved by incorporating OMS. Thus, use of PUNCs for controlled drug release in long term urinary catheterization could be promising in future. Saini et al. investigated the functionalization of silicon urinary catheter by impregnation of bioactive agent macrolide, azithromycin and a fluoroquinolone, ciprofloxacin. Both individual and combination of these drugs helps in the reduction in microbial growth and biofilm formation against *P. aeruginosa* has been revealed in **Figure** **9**. Catheter shows longer antimicrobial durability for four weeks and exhibits a stable real‐time shelf life of one year.^127^

The anti‐biofilm effect of Ag/PU and Au/PU nanocomposites has been investigated with the help of drip flow biofilm reactor and shaker against *E. coli*.^128^ It was concluded that shear forces near the polymer surface greatly influence the morphology and adherence of bacteria to the surface. Swelling and casting methods were used for Ag nanoparticle incorporation into the polymer. During incorporation using swelling method, the Ag nanoparticle solution was poured on the PU film which resulted in swelling of the polymer and penetration of the nanoparticles into it. Similarly, gold nanoparticles were impregnated in the same manner. However, in the casting method, the polymer solution made in tetrahydrofuran was mixed with the AgNP solution under stirring, resulting in the nanoparticle impregnated PU composite. Similar method was followed for Au. Ag modified PU reduced the attachment of live cells by an order of four to seven, whereas Au modified surfaces caused reduction of live bacteria by an order of two to six. The nanoparticles impregnated by the swelling method remain firmly entrapped and bound to the polymer matrix and hence are expected to manifest its antibacterial action for long duration unlike those prepared by coating. Hence, this composite can be readily used for PU catheters.

Enzymes have recently gained special attention as the latest generation of antimicrobials, for example, glucose oxidase, which makes use of glucose to produce hydrogen peroxide (H_2_O_2_) is a well‐known antimicrobial agent.^129^ Recently, Thallinger et al. explored the ability of an enzyme cellobiose dehydrogenase (CDH) to produce H_2_O_2_, which can aid in anti‐biofilm functionalization of urinary catheters.^130^ CDH, an oxidoreductase that functions differently from glucose oxidase, can oxidize cello‐oligosaccharides as well as other oligosaccharides to produce H_2_O_2_ which inhibits growth of bacteria. CDH in the presence of just 1 × 10^−3^ m cellobiose was able to completely inhibit formation of biofilm by *S. aureus*. Another interesting feature of CDH/cellobiose system seen was its ability to oxidize enzymatically hydrolyzed *E. coli* and *S. aureus* EPS, leading to production of H_2_O_2_. It can also be incorporated into the lubricant which is used to minimize discomfort during catheterization.

Increasing resistance of antibiotics on indwelling urological devices has contributed to enhanced rates of nosocomial infections. Thus, the use of bacteriophages as antimicrobials could be a prudent step. Applications of phages for biofilm control hold immense potential. The ability of the phages is to replicate at the infection site, thus growing in numbers where they are most needed. Some phages secrete enzymes that can effectively be used to degrade the EPS matrix of a biofilm.^131^, ^132^ Bacteriophages are like viruses that can selectively infect bacteria, disrupt normal bacterial metabolism, cause the bacterium to lyze rapidly. Infections associated with indwelling urological devices and CAUTIs due to biofilm formation can be prevented using lytic bacteriophages. The use of lytic bacteriophages against well‐known biofilms of *P. mirabilis* and *E. coli* has been described, where 99.99% reduction of biofilm populations was observed. Half of the infection cases are complicated by severe encrustation and blockage at the catheter site, particularly when it occurs due to *P. mirabilis* and in such cases, the only effectual treatment for CAUTIs is the removal of the catheter.^133^ But, Fu et al. have presented the possible applications of bacteriophages to prevent biofilm formation on medical devices, especially for *S. epidermidis* and *P. aeruginosa*.^134^ They coated the sections of Foley catheter with a neutral hydrogel (Bard Lubri‐SilTM) and treated them with bacteriophage cultures. The results unraveled reduction up to 90% in biofilm generation on bacteriophage‐treated French Foley catheters in comparison to the untreated ones.

### 3.4 Blending Process

Blending approach is completely different than the surface functionalization and coating process as the bioactive component is mixed with the material used for catheter development and subsequently processed into a tubular catheter. This leads to the dispersion of the bioactive agent throughout the matrix besides the surface. Here, the compatibility of the bioactive agent with the base matrix is very important so that the mechanical strength of the catheter is protected. Triclosan, 2,4,4′‐trichloro‐2′‐hydroxydiphenyl ether is another World Health Organization approved bioactive agent with low toxicity and a broad range of activities as bactericidal components which can be used to inhibit the catheter infections in controlling infection.^72^ The process involves the melt extrusion of the polymer with required amount of triclosan under appropriate conditions to develop infection resistant catheters. Thome et al. developed low density polyethylene catheters by mixing 0.1%, 0.5%, 1.0%, and 1.5% triclosan and extruding it at 160–180 °C. It was found that the triclosan concentration of 0.5% was enough to impart antimicrobial nature to the catheter.^42^ It was observed that the biofilm formation was also lower with increasing triclosan concentration. Triclosan in combination with PVA has also been observed to be excellent composition for the antimicrobial nature in polyurethane catheters.^135^ PVA helps in the triclosan release due to the swelling of the coated layer in aqueous medium so that the bioactive component may diffuse out of the swollen hydrogel layer. One of the problems with the blending approach for antimicrobial materials development is that the incompatibility arises which leads to the phase separation and hence loss of integrity of the blended matrix. Moreover, a significant amount of the bioactive component is wasted due to its presence in the bulk matrix. A very small fraction stays on the catheter surface and hence the efficiency of the catheter in infection control diminishes.

### 4 Futuristic Approaches for Preventing CAUTIs

**Figure 10:**
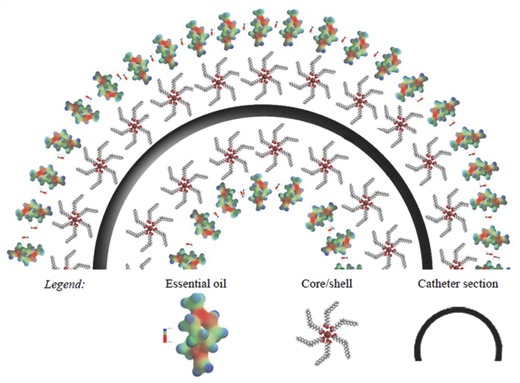
Core/shell/coated‐shell nano‐biosystem (transversal section). Reproduced with permission.^138^ Copyright, 2012, Springer.

Natural antibiotics and antimicrobial agents are produced from products directly harnessed from nature. They are much safer than synthetic biomaterials and work by boosting the body's defense. For example, plant essential oils are obtained from nonwoody parts of vegetables as foliage, through steam or hydrodistillation. They are complex mixture of terpenoids as monoterpenes (C10) and sesquiterpenes (C15) with a variety of aromatic phenols, oxides, ethers, alcohols, esters, aldehydes, and ketones and find use as antimicrobial and insecticidal agents. In the 19th century, eucalyptus oil had been used to clean urinary catheters in hospitals as a precautionary measure to kill bacteria.^136^ The potency of eucalyptus essential oil has been explored against a thick biofilm of *P. mirabilis* on urinary catheters.^137^ The catheters incubated with *P. mirabilis* culture with eucalyptus essential oil at sub‐minimum inhibitory concentration exhibited high anti‐biofilm effect. This effect is based on the potential of eucalyptus essential oil to exhibit high inhibitory effect on the formation of bacterial biofilm. To observe anti‐biofilm effect, catheters were prepared and incubated in tryptic soy broth (TSB) media with *P. mirabilis* ATCC 7002 culture (100 µL mL^−1^), with and without the eucalyptus oil at sub‐MIC concentration (200 µL mL^−1^) and were incubated for 96 h. The SEM analysis confirmed more than 90% reduction in the biofilm formation by *P. mirabilis* in the catheter with eucalyptus oil as compared to the one without oil, which strongly supported the high anti‐biofilm activity of the oil. The antimicrobial activities of several essential oils, namely, tea tree oil, terpinen, cineole, and eugenol against bacteria involved in CAUTIs have been examined.^137^ The activity of these agents against both planktonic cells and biofilms was tested in artificial urine. Chifiriuc et al. developed a different approach by combining the properties of nanoparticles with the antimicrobial activity of the *Rosmarinus officinalis* essential oil.^138^ Here, magnetic nanoparticles (Fe_3_O_4_) with a diameter up to 20 nm were synthesized by precipitation method with microwave conditions and oleic acid as surfactant. Coating on catheter pieces was then carried out with suspended core/shell nanoparticles (Fe_3_O_4_/oleic acid:chloroform), by applying a magnetic field on nanofluid with subsequent adsorption of chloroform diluted essential oil in a secondary covering treatment, as depicted in **Figure** **10**. This nanosystem could be pelliculized on the surface of catheter pieces to provide protection from microbial colonization by *C. albicans* and *C. tropicalis* clinical strains. The CFU counting drastically reduced from 85% to 98% as compared to the uncoated surfaces.

Presently, herbal based natural nanoantibiotics are proving to be an effective substitute to synthetic drugs. Natural drugs bolster the onset of the body's own immune system and triggers it to produce specific protein antibodies that attack harmful pathogens and immobilize them before the onset of disease. Antimicrobial activity of three kinds of commercially available montmorillonite nanoclays including a natural one (Cloisite Na+) and two organically modified ones (Cloisite 20A and Cloisite 30B) against four pathogenic bacteria, i.e., *S. aureus* and *Listeria monocytogenes*, and two gram‐negative ones such as *Salmonella typhimurium* and *E. coli* O157:H7 has been carried out.^139^ In addition, protein based nanomaterials such as AMPs also exhibit anti‐biofilm efficacy. AMPs derived from lactic acid, i.e., lantibiotics like nisin, mersacidin, and nonlantibiotics like pediocin, PA1, enterocin AS48 in the nanomolar range show activity against broad‐spectrum gram‐positive bacteria.^140^, ^141^ Recently, antiadhesive AMP coating was used for the modification of PU catheter. Initially, PU surface was modified by plasma treatment, then polymer brushes were created, and finally antimicrobial peptides are coated. The tethered peptides on the PU catheter surface displayed broad spectrum antimicrobial activity and showed long term activity in vitro and in vivo. The AMP‐brush coating also showed good biocompatibility with bladder epithelial cells and fibroblast cells in cell culture.^142^

Anghel and Grumezescu discovered that *Mentha × piperita*, an essential oil, combined with a ferric chloride/carbon core/shell nanosystem can drastically improve surface exhibiting antiadherent and anti‐biofilm properties against *S. aureus*, *E. coli*, *C. albicans*, *Acinetobacter baumanii*, *E. faecalis*, *K. pneumonia*, and *S. marcescens*.^143^ Nanomaterials of microbial origin such as bacteriophages are being used as therapeutics since a long time to fight against antimicrobial resistance. Carson et al. discovered that Foley catheters coated by a hydrogel treated with a cocktail of bacteriophage cultures were not only capable of preventing biofilm formation by bacteria during CAUTIs but were also able to eradicate >99.9% of an established *E. coli* biofilm.^20^ Plant extracts have been traditional components of antimicrobial technology and biofilm inhibitions.^144^, ^145^ Curcumin, herbal constituent of turmeric powder (*Curcuma longa*) has been a traditional bioactive compound and has been investigated against catheter related infections.^146^ A significant reduction in microcolonies was observed on the biofilms of curcumin treated uropathogens as compared to the untreated surfaces. At the curcumin content of 100 µg mL^−1^, the biofilm was dislodged by 52%, 89%, 76% in *E. coli*, *P. Mirabilis*, and *S. marcescens*, respectively. Interestingly curcumin was found to be very effective in disrupting the matrix (preformed) biofilms of uropathogens and decreasing its thickness. This is evident from the fact that the thickness of *E. coli* biofilm mass is reduced from 16 to 10 µm, while in *P. mirabilis*, it was 11 µm as compared to 6.36 in curcumin treated biofilm. Also, Das et al. designed a sunflower oil modified magneto‐thermoresponsive hyperbranched polyurethane (HBPU)/Fe_3_O_4_ nanocomposite by an in situ polymerization technique and the results obtained showed that desirable interfacial interactions exists between superparamagnetic Fe_3_O_4_ and HBPU as well as the incorporation of Fe_3_O_4_ in HBPU significantly improved antibacterial activity, biocompatibility, and biodegradability in comparison to the native system.^147^

## 5 Conclusion

This review draws attention of the researchers toward the dire need to understand the prevalence and morbidity of CAUTIs and recent advancements in the development of antimicrobial catheters using various therapeutic approaches of which the application of nanomaterials has been vital. Looking ahead with the current scenario, it is lucid that even the development of powerful antibiotics has failed to surmount the critical problem of antibiotic resistance which has already been attempted by pharmaceutical companies, and thus a sharp turn toward the use of nanomaterials has gone into force. There may be various technological approaches in terms of material and bioactive components to control the infection, but a precise approach is very much needed in terms of durability of the system against microbes.^148^ The most appropriate approach would be to inhibit the adhesion of microbes on the catheter surface. Both the bacteriostatic and bactericidal approaches may be taken up to design antimicrobial materials, and the surface which should provide antifouling behavior against microbes. However, it seems that a single strategy would not be effective for the catheter related infection control. The approach should be a combination of the microbial antiadhesion along with the antibacterial behavior against a wide spectrum of microbes. Such an approach has been expected by using a combination of PEG with a large number of bioactive components which would offer bactericidal features.

An important criterion for the antimicrobial catheter is to design the surface where the release of the bioactive component proceeds for a longer duration and at a constant rate. It seems that hydrogels may be visualized as the unique matrix where a slow and sustained release of the agent could take place by diffusion across the swollen matrix. Certainly, the proper combination of the hydrogel matrix and the bioactive agent would be a prime requirement with specific consideration of the biocompatibility and the inflammation at the contact site. Nanosilver in combination with nanogels shows promising future. Furthermore, application of natural antimicrobial agents such as essential oils, AMPs, and curcumin can lead to the prevention of biofilm at the catheter site. The world of antimicrobial catheter development is still wide open and needs coherent efforts to bring in material scientists and biologists together on single platform. Intensive research and relevant clinical trials on modified catheter surfaces to restrict the bacterial migration in the catheter lumen and colonization along the external surface of catheter without causing discomfort to the patient are prudent.

Looking at the existing scenario in the development of infection resistant catheter, what we realize is that cumulative options are limited. The problems have been a serious setback in the healthcare industry due to the level of infection and multidirectional complications in the human body. The “infection” as a word seems to be very simple but could be a very fatal scenario if not taken care by using appropriate research materials in conjugation with cumulative approaches. The developments of functional biomaterial which may offer complete infection control do not show much option and most of them are merely limited more toward the developmental or research stages. Here, we have realized that it is important to have proper combination of material as well as the therapeutic approaches so that a catheter with well‐defined and precise characteristics may be developed. This accomplishment needs a proper coordination between material scientists, biotechnologists, and medical fraternity to join hands together for the interest of mankind.

## Conflict of Interest

The authors declare no conflict of interest.
